# Relationship between ALZpath pTau217 and cognitive test performance in a primary care sample

**DOI:** 10.1002/alz70856_107422

**Published:** 2026-01-09

**Authors:** Robin C. Hilsabeck, Nusha Kheradbin, Liliana Guadagno, Scott Selinger, Heather E Cuevas, Claire Harrison, Joshua T Chang, Ling‐Yu Chang, Tomequa Sears, Ryan Huebinger, Teodora Stoica, Carolynne Holbrook, Michael Metke, Donald Biehl, Lee Honigberg, Andreas Jeromin

**Affiliations:** ^1^ UT Health San Antonio, San Antonio, TX, USA; ^2^ Dell Medical School at The University of Texas at Austin, Austin, TX, USA; ^3^ UT Austin Dell Medical School, Austin, TX, USA; ^4^ UT Austin School of Nursing, Austin, TX, USA; ^5^ University of Texas Southwestern, Dallas, TX, USA; ^6^ ALZpath, Carlsbad, CA, USA; ^7^ Neurocode, Bellingham, WA, USA; ^8^ Atlantic Biomarkers, Alachua, FL, USA

## Abstract

**Background:**

Blood biomarkers are a promising tool for identifying Alzheimer's disease (AD), particularly in primary care settings where AD and related dementias are frequently undiagnosed. More information in real‐world clinical cohorts is needed to increase confidence in the utility of plasma biomarkers, particularly phosphorylated tau at threonine 217 (pTau217), which has demonstrated reliability in indicating AD pathology (Devanarayan V, et al., 2024) and strong correlations with positive amyloid PET scans (Ashton N, et al., 2023). This study aimed to examine the relationship of plasma pTau217 to cognitive test performance in a real‐world primary care cohort.

**Method:**

Sixty‐eight older adults completed the Quick Mild Cognitive Impairment (Qmci) screen during a routine primary care visit and a blood draw on the same day or within six weeks. Participants with elevated ALZpath pTau217 levels (> 0.63 ng/L) were classified as positive, and those with pTau217 levels between 0.34‐0.63 ng/L were classified as intermediate (Mammel AE, et al., 2025). A subset of participants (*n* = 58) completed a neuropsychological test battery (NTB) consisting of language, memory, and executive functioning (EF) measures. Participants were considered cognitively impaired if they had two or more demographically adjusted T scores < 40 within the language, memory, or EF domains or one demographically adjusted T score < 40 in each of the three domains.

**Result:**

Average age was 68.8 years (69% female, 90% White), and 26% were APOEε4. Five participants (7%) had positive pTau217 levels, and 15 (22%) had intermediate levels. Of the five pTau217 positive participants, none were cognitively impaired on the Qmci, but three were cognitively impaired on the NTB, two in the memory domain and one in memory and EF. Of the 15 with intermediate pTau217 levels, two were impaired on the Qmci and four were impaired on the NTB, all in the memory domain only. Higher average concentrations of pTau217 correlated significantly with lower composite memory T scores (*r* = ‐.26; *p* = 05) but not global, language, or EF T scores.

**Conclusion:**

Elevated levels of pTau217 were relatively low in this generally cognitively intact primary care cohort, but showed adequate concordance with cognitive performance, especially memory.

